# Potential Zoning of Construction Land Consolidation in the Loess Plateau Based on the Evolution of Human–Land Relationship

**DOI:** 10.3390/ijerph192214927

**Published:** 2022-11-13

**Authors:** Minjuan Lv, Zhiting Chen, Lingling Yao, Xiaohu Dang, Peng Li, Xiaoshu Cao

**Affiliations:** 1 School of Geography and Tourism, Shaanxi Normal University, Xi’an 710119, China; 2 Academy of Natural Resources and Territorial Space, Shaanxi Normal University, Xi’an 710119, China; 3 Key Laboratory for Urbanization and Land Environment Geo-Simulation in Northwest China, Shaanxi Normal University, Xi’an 710119, China; 4 Loess Plateau Observation Station of Coupled Human and Natural System, Shaanxi Normal University, Xi’an 710119, China; 5 College of Geology and Environment, Xi’an University of Science and Technology, Xi’an 710054, China; 6 State Key Laboratory of Eco-Hydraulics in Northwest Arid Region of China, Xi’an University of Technology, Xi’an 710048, China

**Keywords:** rank-size, allometric scaling, consolidation potential zone, the Loess Plateau

## Abstract

Towns serve as the basic unit of implementation for comprehensive land consolidation and rehabilitation. The utilization of scaling law can provide a new perspective for construction land consolidation. From two perspectives of the town hierarchic system and the growth of a single town, this research applies the Rank-Size Rule and Allometric Scaling Law to analyze the scale structure, hierarchy and allometric scaling evolution relationship of population and construction land in the Loess Plateau at the town scale in 2000, 2010, and 2017. Furthermore, the consolidation potential of construction land is divided into five zones and puts forward recommendations for the comprehensive consolidation of the construction land. The results indicate: (1) The majority of towns have small or medium populations and 62% of the towns in the study show negative population growth. Geographically, the northern part has a smaller population size compared with the southern part. 96% of the towns show an increasing trend in the quantity of construction land, and the south and north parts of the study area have more construction land compared with the center part. The zone of the Valley Plain has the largest population size, and the zone of the Sandy and Desert Area has the largest quantity of construction land. (2) The rank-size distributions of both population and construction land comply with the power-law relation. The population hierarchy has changed from equilibrium to concentration, while the hierarchy of construction land shows an opposite pattern. So, the whole town hierarchic system of the Loess Plateau is gradually tending to the optimal distribution, which is the town hierarchic system gradually forming an ideal sequence structure. (3) The population-construction land relationship obeys the allometric scaling law, and the major allometric type is positive allometry. The human–land relationship tends to be coordinated, and the town system tends to be reasonable. The allometric scaling coefficient is not robust in different geographical areas, especially in Irrigated Agricultural Areas. Furthermore, 90% of the towns have weak coordination of human–land relationships, and 60% of the towns have a relatively faster growth rate of construction land than the relative growth (decline) rate of population. (4) The consolidation potential of construction land is divided into five types. High Consolidation Potential Area concentrates in the Eastern Loess Plateau, while Medium and Low Consolidation Potential Area concentrically distribute in the Western Loess Plateau. The Human–land Coordination Area has a small number and scattered spatial distribution. The land use of towns that are concentrated around prefecture-level cities or with unique resources is not intensive enough. The zoning of construction land consolidation potential based on the results of the allometric scale is in line with reality, and local governments should make use of the characteristics and trends of the town system to formulate planning schemes to promote the integrated development of urban and rural areas.

## 1. Introduction

Under the process of urbanization and industrialization, the urban construction land has continued to expand with a large number of rural residents moving to urban areas [1]. However, the rural construction land has not decreased correspondingly but shows a trend of increment with reducing population and outward expansion with internal vacancies [2,3], leading to a severe contradiction between humans and the land. In December 2019, the Natural Resources Ministry issued a notice on carrying out the pilot work of comprehensive land consolidation and rehabilitation ([2019] No. 194) with towns as the basic implementation unit. It is the general trend to further vacate idle construction land and improve the efficiency of construction land use. The key to the problem is how to optimize the scale and distribution of construction land and improve the land-use efficiency and intensity under the premise of complying with the town hierarchic system and human–land coordinated relationship [4,5,6,7].

Land consolidation generally includes agricultural land consolidation and construction land consolidation [8]. The research mainly focuses on the connotation, zoning of consolidation type [9], consolidation timing sequence [10,11,12] and its impact on agricultural production [13], social economy [14,15,16,17] and ecological environment [18,19,20,21,22]. Due to different social development backgrounds, foreign scholars focus more on the study of agricultural land consolidation [23,24,25,26,27,28,29]. In terms of research content, the existing research on construction land consolidation has not paid attention to the town hierarchic system, but only analyzes the situation of a single research unit. The purpose of taking towns as the basic implementation unit of land consolidation is to promote the integrated development of urban and rural areas. One of the signs of regional urban–rural integration is the integration of town system structure [30]. In terms of research methods, several different methods are universally used to estimate the consolidation potential of construction land, including the per capita/household construction land method [31], the idle rural residential land method [32], and the comprehensive evaluation method [33,34]. First and foremost, the potential assumption of using Per Capita Indices is that the quantity of construction land and population size are linearly-related. However, due to the prevalence of economies of scale and the agglomeration effect, the relationship between population and the change in the quantity of construction land is nonlinear [35,36]. It has been proven theoretically that the proportion between population and construction land is 0.85 [37], which is a reasonable phenomenon. So, there is a problem in overestimating the consolidation potential by this method [38]. Secondly, the idle rural residential land method is always used for specific villages or counties [32], and the consolidation potential is determined by calculating the idle proportion but cannot be generalized to a regional scale. Therefore, this method limits the analysis at the medium and macro scales. Thirdly, the comprehensive evaluation method focuses on the classification of the consolidation potential of construction land, and comprehensively considers the comprehensive role of natural, social, economic and other factors [39]. The studies based on these methods all consider a certain time node and fail to consider the impact of spatio-temporal change characteristics and evolution trends on the potential scale and spatial pattern of construction land consolidation. From the perspective of research objects, most studies on the regional scale take provinces, cities and counties as research units, while studies on smaller research units are relatively few. However, relatively independent and complete basic geographic units can be accurately divided at a more microscopic level, and the internal structure can be more clearly presented.

Based on the above analysis, in order to reasonably zone the potential of construction land consolidation, it is necessary to analyze its spatial distribution, hierarchical structure and dynamic evolution characteristics. Therefore, the Rank-Size Rule and the Allometric Scaling Law are applied for analysis, and the allometric scaling coefficient can be derived from the Zipf coefficient. The Rank-Size Rule can reflect the agglomeration or dispersion characteristics of population and construction land variables [40] and judge the spatial hierarchical structure characteristics of the town system. Allometric scaling law was first introduced in the field of biology by Naroll et al. [41], and then developed and improved by Bettencourt [42,43], Batty [44,45], and Chen [46] to describe the relative growth rate between two different elements or of a single element relative to the whole. Allometric scaling can be divided into two basic categories: cross-sectional allometry and longitudinal allometry. Cross-sectional allometry describes the static human–land pattern of the town hierarchic system, and the population-area allometric scaling relationship is the most studied [46,47]; while longitudinal allometry describes the dynamic process of human–land relationship in time evolution [30], for example, Chen [48] studied the human–land allometric relationship in Xinyang City, Henan Province, and pointed out that the reason for the abnormal scale is a large number of “city building movement”. There are few studies on the type of division or zoning of regions based on the results of allometric scaling, Hong et al. [49] classified the type of human–land coupling based on the results of allometric growth and proposed that Guangzhou should pay attention to preventing and controlling the extensive growth of construction land in the outside ring.

Understanding and using the scaling law to guide the consolidation of construction land is crucial for the coordinated development of human–land relationships. This paper takes towns as the evaluation unit to analyze the evolution of the human–land relationship and construction land consolidation in the Loess Plateau in 2000, 2010 and 2017. This paper first analyzes the spatio-temporal evolution characteristics of population size and construction land quantity, and secondly explores structure characteristics of the town hierarchic system based on the Rank-Size Rule. Thirdly, we investigate the cross-sectional allometry relationship and longitudinal allometry relationship using the Allometric Scaling Law. Last but not least, based on the allometry results, this paper divides the potential zones of construction land consolidation and puts forward consolidation suggestions. Relevant conclusions can provide the basic basis for the human–land coordinated development and the optimization of town structure in the Loess Plateau.

## 2. Materials and Methods

### 2.1. Study Area

**Figure 1:**
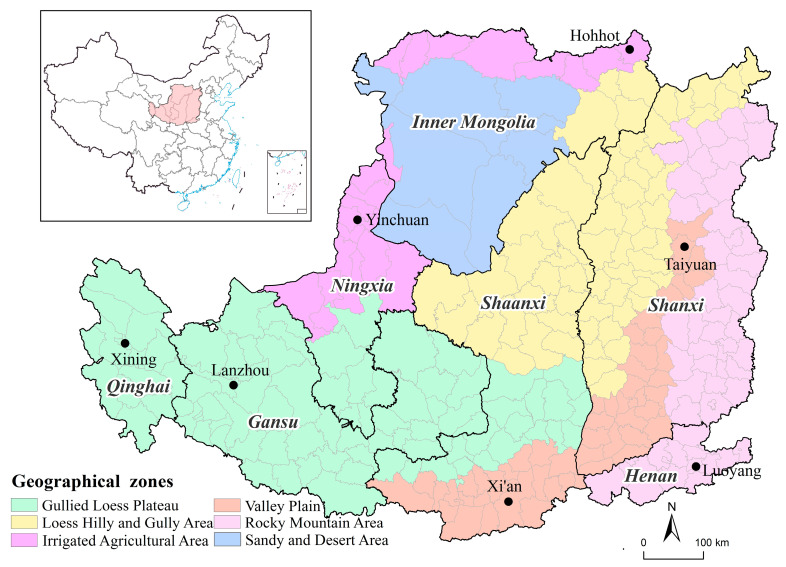
Geographical zones of the Loess Plateau.

According to the demarcation defined by the Comprehensive Survey Team on the Loess Plateau of the Chinese Academy of Sciences (CSTLP-CAS), the Loess Plateau is located between 33°41′ N–41°16′ N, 100°54′ E–114°33′ E, mainly including all Shanxi Province and Ningxia Autonomous Region, most of Shaanxi Province, and partially Henan, Gansu, Qinghai Provinces and the Inner Mongolia Autonomous Region, with 284 county-level administrative units and a total area of 635,000 km^2^. The Outline of Comprehensive Management Plan for the Loess Plateau Area (2010–2030) further divides the Loess Plateau into six geographical zones to better reveal its spatial differences and regional differentiation characteristics, including Valley Plain, Loess Hilly and Gully Area, Gullied Loess Plateau, Irrigated Agricultural Area, Sandy and Desert Area and Rocky Mountain Area (Figure 1).

### 2.2. Research Data

The main sources of the research data in this paper are as followings. (1) The research unit of this research is the town, with a total number of 3001. The boundaries of the towns in this paper are derived from the administrative division published by the Ministry of civil affairs in 2017, excluding the towns where county seats are located and combining towns according to the “Announcement on the Withdrawal and Merger of Towns and the Adjustment of Administrative Division” of each province. (2) The data on the permanent resident population are from the fifth and sixth Demographic Census and Statistical Yearbook 2018 (which shows the data for the year 2017) of the seven provinces and regions. (3) The construction land in this paper is derived from the 30m-resolution Land Use Data published by Resource and Environment Science and Data Center, Chinese Academy of Sciences (http://www.resdc.cn, accessed on 26 September 2021).

### 2.3. Research Methods

#### 2.3.1. Rank-Size Rule

The rank-size rule measures the size distribution of a regional urban system by the relationship between the size of the city and the rank of the city size [47]. This paper assumes that the population size and its rank at the town scale in the Loess Plateau obey the following power law:

(1) $$P_{i}=P_{1}\times R_{i}{}^{-q}$$

(2) $$\ln P_{i}=\ln P_{1}-q\ln R_{i}$$

where $P_{i}$ and $R_{i}$ represents the size (or quantity) and the rank of the population (or construction land) of Town i, respectively; $P_{1}$ represents the population size (construction land quantity) of the town with the first rank. q represents the Zipf coefficient, which is a constant value: when q=1, the population size (construction land quantity) system is in the optimal distribution in the natural state; when q>1, it is the concentrated distribution; when q<1, it is the equilibrium distribution.

#### 2.3.2. Allometric Scaling Law

In China, Chen Yanguang [37,50] has conducted a lot of research on the allometric scaling law and proposed that it reflects the character that two connected elements in one region change according to a constant ratio. Its mathematical model can reflect a clear structural relationship, and the allometric scaling law can be expressed as follows:

(3) $$A_{\left(t\right)}=aP_{\left(t\right)}{}^{b}$$

where $A_{\left(t\right)}$ represents the quantity of construction land of a town at the time t; a is the scale coefficient; $P_{\left(t\right)}$ represents the population size of the town at the time t, b is the allometric scaling coefficient. Take the logarithm of both sides of Equation (3) at the same time to obtain [43]:

(4) $$\log A_{\left(t\right)}=b\times \log P_{\left(t\right)}+\log a$$

Equation (4) is a linear function. Therefore, the logarithm of construction land and population size can be used to fit the linear function, and the slope of the fitting line is the scale factor b.

A large number of experimental observation data show that the allometric scale coefficient of population and land is about 0.85 [37,46,50], regardless of longitudinal allometry or cross-sectional allometry. Due to the existence of negative growth of population size at the town scale, there could be a negative value in the allometric coefficient. When |b| = 0.85, the population size and the quantity of construction land maintain the same changing speed and have a linear relationship; when |b| > 0.85, there is a positive allometric, which is a super-linear relationship, indicating the relative growth rate of construction land quantity is higher than that of population size; when |b| < 0.85, there is a negative allometric, which is a sub-linear relationship, indicating the relative growth (decline) rate of population size is faster than that of construction land quantity. When the population expands at the Positive Allometry I, II and III, and the Negative Allometry I and II, both population size and construction land expand at the same time. When the population shrinks at the Positive Allometry I, II and III, and the Negative Allometry I and II, the population shrinks when the construction land increases (Table 1). With the lower coordination of the human–land relationship, the more mismatch between the growth rates of construction land and population and the higher the urgency of consolidation.

## 3. Results

### 3.1. The Characteristics of Structures and Changes in Population Size and Construction Land

#### 3.1.1. More Than Half of the Town Population Has Decreased, and the Overall Spatial Feature of Population Size Is “Small in the North and Large in the South”

**Figure 2:**
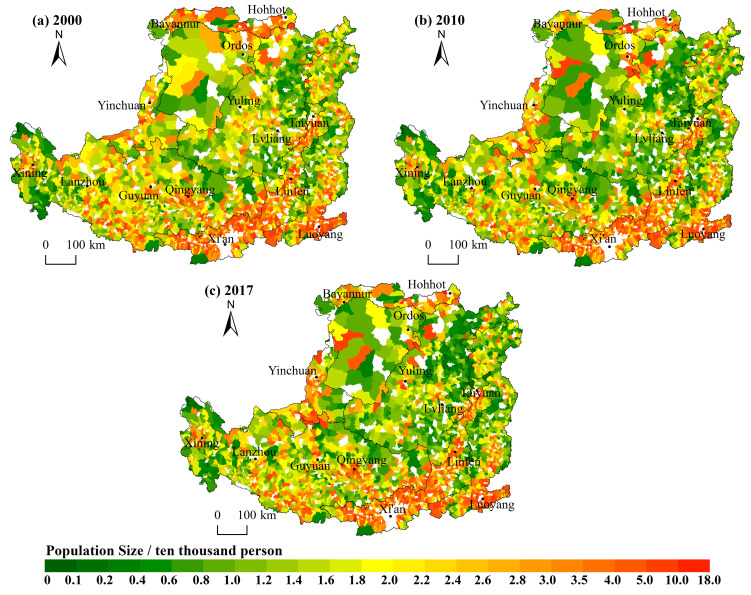
Spatial patterns of population in the Loess Plateau, 2000–2017. The white patches in the picture are the towns where the sub-district and county seats are located.

Over the past 17 years, nearly 62% of the town’s population has decreased, leading to the serious problem of population loss. The largest population of permanent residents increased from 144,501 to 171,342, and the smallest population decreased from 1255 to 355. The population size shows the following features in spatial distribution. Firstly, the distribution of population size had remained a pattern of “small in the north and large in the south”, decreasing from the southeast warm temperate semi-humid areas with good natural conditions to the northwest semi-arid and arid areas. The towns with larger populations are mainly concentrated in the Weihe Plain, Fenhe Plain, Hetao Plain and the lower reaches of the Yiluo River, forming Taiyuan Urban Agglomeration, Guanzhong Plain Urban Agglomeration and Ningxia Urban Agglomeration along the Yellow River. Towns with a small population are mainly located in the Lvliang Mountains, Taihang Mountains, Ordos Plateau and the border area between Shaanxi, Gansu and Ningxia. Secondly, the distribution of town population sizes in each zone shows a concentric circle decreasing outward with cities (or county-level cities) as the center, and the pattern is evident where Xi’an, Taiyuan and Luoyang act as the cores (Figure 2). Thirdly, the spatial pattern of the average annual growth rate is basically consistent with the pattern of population size. The towns with reduced populations are mainly in the mountainous areas with poor natural geographical locations, less-developed economies and traditional agricultural and animal husbandry-dominated areas, such as the Lvliang Mountain area, Taihang Mountain area, and Ordos Plateau.

#### 3.1.2. The Overall Quantity of Construction Land Increased and the Spatial Feature Is “Large in the South and North but Small in the Middle”

**Figure 3:**
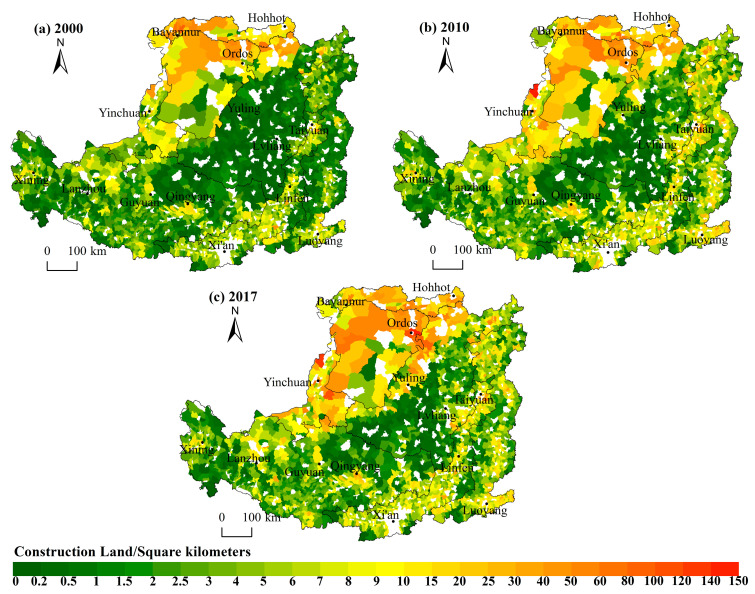
Spatial patterns of construction land in the Loess Plateau, 2000–2017.

**Figure 4:**
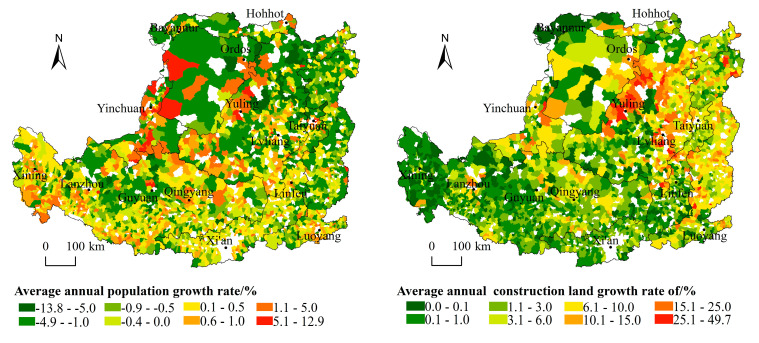
Annual average growth rate of population and construction land in the Loess Plateau, 2000–2017.

From 2000 to 2017, the construction land in all towns increased. The largest construction land among the towns increased from 65.57 km^2^ to 149.21 km^2^, and the smallest one also increased from 0.004 km^2^ to 0.021 km^2^. Meanwhile, the construction land per capita increased from 173.56 m^2^ to 314.30 m^2^. The construction land shows the following features in spatial distribution. Firstly, the quantity of construction land shows an overall distribution characteristic of “larger in north and south while smaller in the middle”. The towns with large amounts of construction land are mainly distributed in the zones of Sandy and Desert Area and Irrigated Agricultural Area in the north of the Loess Plateau where the land is vast but low in land-use intensity. Secondly, the zone of the Valley Plain, and the Qin River Valley and the plain in the lower reaches of the Yiluo River in the zone of the Rocky Mountain Area have flat land and a concentrated population with a large amount of construction land. The towns with small quantities of construction land are mainly distributed in the Loess Hilly and Gully Area and the Gullied Loess Plateau. Constrained by topographic conditions, these towns occupied a small piece of land with a scattered and sparse population and a correspondingly small amount of construction land (Figure 3). Thirdly, the most evident growth in construction land occurred in the Golden Triangle of Shanxi, Shaanxi and Inner Mongolia where the development of the industry of coal and other resources promoted the rapid growth of construction land. The second largest growth occurred in the Lvliang mountain area and Taihang mountain area (Figure 4).

### 3.2. The Rank-Size Characteristics of Population Size and Construction Land

#### 3.2.1. The Population Size Hierarchy Tends to Equilibrium Distribution, and the Major Town Population Sizes Are Small and Medium-Sized

**Figure 5:**
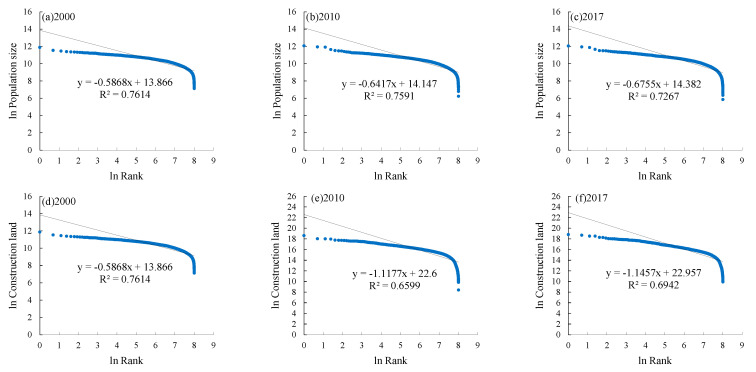
Ln-ln graph of rank-size distribution of population and construction land, 2000–2017.

The Zipf coefficients of population size from 2000 to 2017 were less than 1, which means the population size hierarchy tends to equilibrium distribution and the town-size hierarchy system is not perfect. That is, there was a large number of towns in the middle and low order but a relatively low number of towns in the high order. In 2017, the proportion of towns with a population of less than 30,000 in the Loess Plateau reached 78.8% (Figure 5).

**Figure 6:**
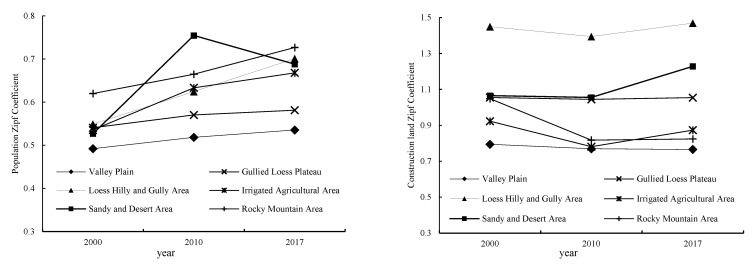
Dynamics of Zipf coefficient of population and construction land, 2000–2017.

In terms of geographical zones, consistent with the regional results, the Zipf coefficient of each zone is also less than 1, and the sequence of the Zipf coefficients of the six zones in 2017 from high to low is the Rocky Mountain Area (0.7267), Loess Hilly and Gully Area (0.701), Sandy and Desert area (0.688), Irrigated Agricultural Area (0.6679), Gullied Loess Plateau (0.5813) and Valley Plain (0.5355). In the zone Rocky Mountain Area, the Qin River Valley and the plain in the lower reaches of Yiluo River have a large population size, while the population size of the Taihang mountain area is small, leading to the evident difference between the north and south where the Zipf coefficient is the largest. There is little difference in natural conditions in the zone of Valley Plain, so the population size of the towns is highly balanced with the smallest Zipf coefficient. Compared with 2000, the Zipf coefficients of the six zones increased by 0.11, 0.15, 0.16, 0.13, 0.04 and 0.04, respectively, indicating that the population is concentrated in high-level towns and the town-size hierarchy system is gradually improving (Figure 6). The zone of Loess Hilly and Gully Area increased the most due to the population increment and concentration in the surrounding towns of Yulin caused by the economic diffusion effect brought by the development of the national energy-chemical industry base in North Shaanxi in 2000.

#### 3.2.2. The Construction Land Hierarchy Tends to Concentrate Distribution, and the Majority of Towns Are Large-Sized

The Zipf coefficients of construction land from 2000 to 2017 were larger than 1 but close to 1, which means the construction land hierarchy tends to concentrate distribution and the town-size hierarchy system is more reasonable. Comparatively, the construction land Zipf coefficients are larger than the population Zipf coefficients, indicating the town-level construction land is more concentrated than the population (Figure 5).

By geographical zones, the sequence of the construction land Zipf coefficients in 2017 from high to low is the Loess Hilly and Gully Area (1.469), Sandy and Desert area (1.2285), Gullied Loess Plateau (1.0542), Irrigated Agricultural Area (0.8729), Rocky Mountain Area (0.824), Valley Plain (0.7657). The Zipf coefficients of the Loess Hilly and Gully Area, Sandy and Desert Area and Gullied Loess Plateau are all greater than 1, indicating each of the three zones has significant variation in the amount of construction land because of the different natural and socio-economic conditions. The Zipf coefficients of the Irrigated Agricultural Area, Rocky Mountain Area and Valley Plain are all less than 1, where the towns mostly have a large amount of construction land with small variations between each other, and therefore, the towns are relatively balanced. The construction land Zipf coefficients in the Valley Plain, Irrigated Agricultural Area and Rocky Mountain Area decreased by 0.03, 0.05 and 0.22, respectively, from 2000 to 2017, tending to a more balanced status because of all the towns’ construction land was growing. The construction land in the Sandy and Desert Area is large and growing rapidly with the Zipf coefficient increasing by 0.16, mainly due to the development of coal resources in Ordos and other places leading to the increasing demand for construction land, which further leads to the significant variation in the amount of construction land of between towns. From the perspective of different geographical zones, the town-size hierarchy system based on the construction land is not stable, which further indicates that the construction land in towns was growing in disorder during the rapid urbanization period (Figure 6).

### 3.3. The Allometric Scaling Relationship between Population and Construction Land

#### 3.3.1. The Integrity of the Town System Is Gradually Improved and Gradually Shifts to a More Reasonable Pattern

**Figure 7:**
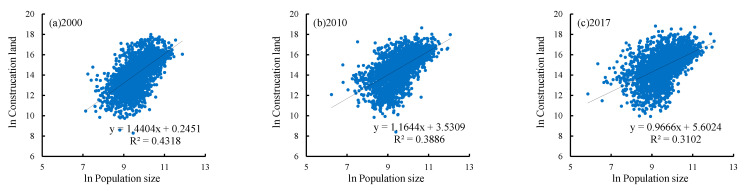
Ln-ln graph of allometry of population-construction land, 2000–2017.

From 2000 to 2017, the allometric scaling coefficient was greater than the theoretical value of 0.85, indicating a stage of positive allometry where the relative growth rate of construction land is larger than that of the population. Meanwhile, the allometric scaling coefficient b decreased to 0.9666 in 2017, which means the human–land relationship in the Loess Plateau is gradually becoming coordinated, bringing about a greater scale effect (Figure 7). According to the analysis above, this change is due to the fact that the town system based on population and construction land has gradually become the optimal distribution in recent 17 years. That is the coordinated development of large, medium and small towns.

To test the robustness of the estimation results, and see whether the coefficient change with different regions, we also estimated the allometric scaling relationship between population and construction land in different geographical zones. The estimation results by geographical zones show that the allometric scaling coefficient is not robust, which is quite different from the estimation of regional samples. Firstly, the estimation results of the Valley Plain, Rocky Mountain Area, Gullied Loess Plateau and Loess Hilly and Gully Area are consistent with the overall change in the region. The human–land relationship in the Valley Plain and Rocky Mountain Area tends to be coordinated, while the Gullied Loess Plateau shows that the relative growth rate of construction land is still growing too fast, and the construction land in the Loess Hilly and Gully Area is relatively scarce. Secondly, the allometric scaling coefficient of the Irrigated Agricultural Area from 2000 to 2017 was less than 0.85, which shows that the town hierarchic system has not yet formed an ideal sequence structure. What is more, it does not show its large-scale economic effect, but indirectly reflects the shortage in construction land resources under the rapid population growth. Third, the allometric scaling coefficient of the Sandy and Desert Area had increased from 2000 to 2017, it was always around 0.85. The human–land relationship has been relatively harmonious (Table 2).

#### 3.3.2. The Major Allometric Type of All Towns Is Positive Allometry, and Most Towns Are with the Weak Coordinated Human–land Relationship

**Figure 8:**
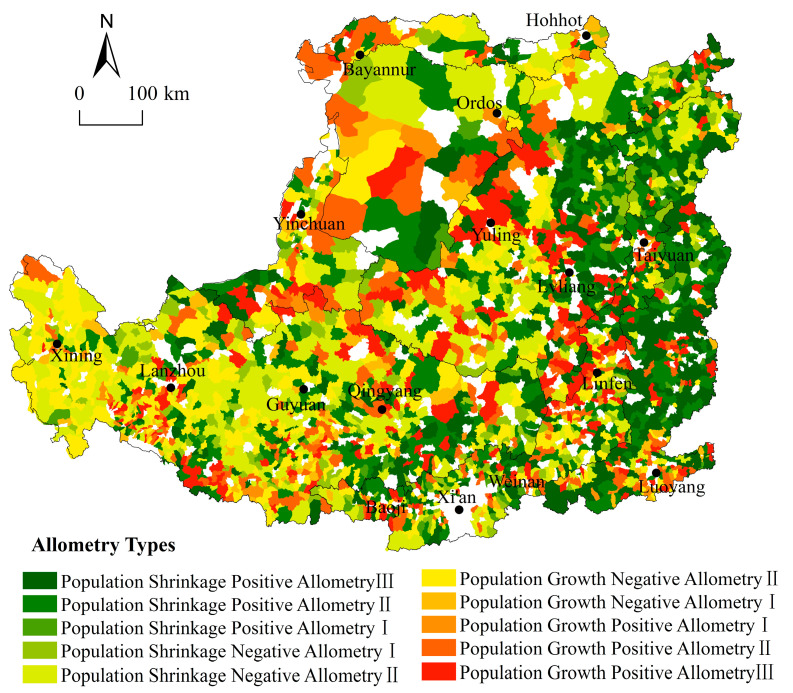
Allometric growth types and levels in the Loess Plateau.

Consistent with the previous results of cross-sectional allometry, in the period from 2000 to 2017, the population and construction land allometric relationship in the 3001 towns in the Loess Plateau is of weak coordination. The ratio of positive allometry to negative allometry is 1.44:1, indicating the relative growth rate of construction land in most towns is higher than the relative growth (decline) rate of population, which further verified the rank-size results of balanced growth of construction land. The above has shown that the allometric scaling coefficient is not robust among the geographical zones. By geographical zones, the main findings are as follows (Figure 8):

(1) The allometry type of the zone of the Gullied Loess Plateau is mainly negative allometry which is dominated by Population Growth Negative Allometry II, Population Shrinkage Negative Allometry II, and Population Growth Negative Allometry I, with each type accounting for 55.32, 51.82 and 48.35% of the towns. The population of the East Qinghai area continues to grow and the speed is faster than the growth of construction land because of the large minority population and animal husbandry. Due to the fragile ecological environment in the Middle Gansu, and the South Ningxia areas, these towns are mainly engaged in the agricultural industry; the government reduces the population mechanically through a series of immigration programs. For example, in 2020, the permanent residence rate of Pengyang County was only 65%. The difference between the natural environmental and government policies has led to a large difference in the human–land relationship within this zone.
(2) The zone of the Loess Hilly and Gully Area is dominated by positive allometry, specifically Population Growth Positive Allometry III (30.14%), Population Shrinkage Positive Allometry I (23.34%) and Population Shrinkage Positive Allometry III (22.78%). This zone has a fractured landscape and topography, and the major types are mainly distributed in the six northern counties of Yulin and Lvliang Mountain, indicating a sharp contradiction between humans and land. The six northern counties of Yulin are the core of the construction of the energy and chemical industry base in Northern Shaanxi, attracting population and industry for agglomeration, resulting in the fast-growing construction land. In 2018, Yulin’s energy and chemical industrial construction land accounted for 26% of Yulin’s total construction land, but its output level was significantly lower than that of industrial parks in the same area, resulting in poor intensive land use. The Lvliang Mountain has a fragile ecological environment and serious soil erosion problem, and the population is gradually migrating outwards. However, the construction land is growing extensively, resulting in an urgent and complicated consolidation.
(3) The dominant allometric types in the zone of the Valley Plain are Population Growth Positive Allometry II (24.33%), Population Growth Positive Allometry III (24.32%) and Population Shrinkage Negative Allometry I (23.53%). The zone is the center of regional economic activities and has formed the Taiyuan Urban Agglomeration and Guanzhong Plain Urban Agglomeration. In general, this zone is experiencing accelerated non-agriculturalization of the rural population and rapid expansion of construction land. With Xi’an, Linfen, Taiyuan and other prefecture-level cities as the core, a series of “city building campaigns” led to a large number of cultivated land being turned into construction land. What is more, it has significant destructive power on the evolution of the town system.
(4) The allometry type of the zone of Rocky Mountain Area is dominated by Population Shrinkage Positive Allometry III and II (accounting for 44.35 and 25.78% of the towns, respectively). The towns of these two allometry types are mainly distributed in the mountainous areas in the middle and north of Taihang Mountain, where hills are widely distributed, and there are few plains. Since Jincheng, Changzhi, Yangquan and other cities have coal resources, since the 1990s, local people have been engaged in agriculture and industry at the same time. For example, the non-agricultural employment rate of the rural population in Qinshui County has been maintained at about 35%. In the context of ecological environment governance, the merger and abandonment of small-scale enterprises led to a large amount of construction land being in an idle state. The construction land is increasing while the population keeps decreasing leading to a notable phenomenon of a “hollow village”, and the outflow population in this zone generally gathers in the county town.
(5) The zone of the Irrigated Agricultural Area is mainly composed of the Ningxia Yellow River Irrigation Areas and Inner Mongolia Hetao Plain Irrigation Areas, within which the water source is sufficient and large oases and agricultural irrigation areas are widespread. The dominant allometry types of this zone are Population Growth Positive AllometryII, and Population Growth Negative Allometry II and I (accounting for 11.33, 7.79 and 8.79% of the towns, respectively). Due to the existence of the urban agglomeration along the Yellow River in Ningxia and the Hohhot–Baotou-Ordo urban agglomeration, the construction land is mounting up steadily, such as the famous “ghost town” phenomenon.
(6) The Sandy and Desert Area is distributed in the hinterland of the Ordos Plateau with the Mu Us Desert being the dominant geomorphic unit. The main allometry types of this zone are Population Growth Positive Allometry I, Population Shrinkage Positive Allometry I and Population Growth Positive Allometry II (accounting for 4.55, 3.08 and 2.67% of the towns, respectively). Due to the development of coal resources, the construction land in Ordos, which borders Shaanxi, has increased significantly and faster than the population.

### 3.4. The Zones of Construction Land Consolidation and Consolidation Suggestions

The above analysis shows that the natural environment has a long-term and relatively stable impact on the overall hierarchy of towns or the growth of individual towns and significant fluctuations in growth are unlikely to occur without mutations. Changes that do not follow the allometric scaling law are mostly related to socio-economic processes, such as the mining of coal resources, urban sprawl resulting from the development of real estate, and population outflow driven by low agricultural output.

**Figure 9:**
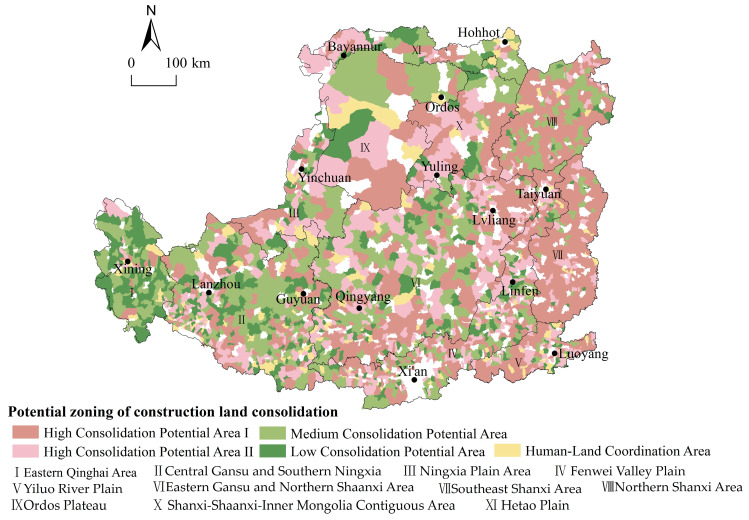
Potential zoning of construction land consolidation in the Loess Plateau.

Based on this, the following two steps are taken to divide the zones of construction land consolidation. Firstly, towns were divided into five zones based on their allometric results, namely High Consolidation Potential Area I, High Consolidation Potential Area II, Medium Consolidation Potential Area, Low Consolidation Potential Area, and Human–land Coordination Area. The specific division method is as follows: High Consolidation Potential Area I comprises Population Shrinkage Positive Allometry I, II and III. High Consolidation Potential Area II comprises Population Growth Positive Allometry II and III. Medium Consolidation Potential Area comprises Population Shrinkage Negative Allometry I and II. Population Growth Negative Allometry II is defined as a Low Consolidation Potential Area. Population Growth Negative Allometry I and Population Growth Positive Allometry I constitute the Human–Land Coordination Area. Subsequently, the six geographical zones were further divided into eleven sub-zones based on the dominant factors of the human–land relationship of each zone analyzed above. The final results are shown in Figure 9.

The High Consolidation Potential Area I is where the relative growth rate of land for construction is higher than the relative rate of decline in population. In areas of this type, the population is decreasing while the land is increasing, resulting in a highly unbalanced human–land relationship. Such areas are primarily located in the eastern part of the Loess Plateau, mainly in Northern Shaanxi and the Southeast. Although these towns have seen a reduction in population, their total population remains large. Therefore, the core of coordinating the human–land relationship in these towns is to strictly prohibit the growth of construction land and reduce population outflow. In addition, the above analysis shows that there is a large amount of abandoned industrial and mining land in these towns. Therefore, the direction of construction land consolidation in areas of this type lies in: firstly, reclaiming the abandoned industrial and mining land as arable land, and actively using the policy of linking urban construction with the reduction in land for rural construction to support the development of the core towns; secondly, strengthening the construction of high standard farmland and promoting moderate scale operation of agriculture, to prevent the further loss of population.

The High Consolidation Potential Area II is where the relative growth rate of built-up land is faster than the relative growth rate of population. Proximity and resource uniqueness is critical for towns of this type. These towns are either distributed in proximity to prefecture-level cities or have unique resources (e.g., coal), primarily found in the Fenwei Valley Plain, Ningxia Plain Area, Yiluo River Plain, Hetao Plain, and the Shanxi–Shaanxi–Inner Mongolia Contiguous Area. These towns have favorable natural conditions and resources, but the scattered expansion of construction land in the early stages of development has led to poor scale effects. The core of coordinating the human–land relationship in these towns is to actively exploit the potential of the stock of construction land, promote the agglomeration effect, and guide the orderly growth of the population. Recommendations for the consolidation of construction land in areas of this type are as follows: firstly, redeveloping inefficient construction land in towns located in the vicinity of prefecture-level cities; secondly, making efficient use of land in industrial parks. To accommodate the transformation of the coal chemical industry, take initiatives such as developing underground space, establishing standard factory buildings and optimizing access standards for enterprises to promote the intensive use of construction land, stimulate the scale effect of construction land, and facilitate population clustering.

The Medium Consolidation Potential Area is where the relative decline rate of the population is higher than the relative growth rate of construction land. Towns of this type are mostly found in central Gansu and southern Ningxia, with a few in eastern Gansu and northern Shaanxi. Given that the major challenges in these towns are the constant loss of population and the relatively small amount of construction land, priority should be given to the following two aspects in the consolidation of construction land: firstly, promoting the consolidation and reclamation of abandoned construction land and abandoned rural residence left behind by people transferred to urban areas to expand the scale management of cultivated land; secondly, developing agriculture-led industries (e.g., dryland terracing construction and the red plum and apricot industries in Pengyang County) and extending the industrial chain to improve the input-output efficiency, which will further promote the return of the population.

The Low Consolidation Potential Area, differing from the other areas mentioned above, has a higher relative population growth rate than the growth rate of construction land. Towns of this type are mainly found in eastern Qinghai. The construction land in the towns in this area is relatively sparse and is expected to increase moderately.

In the Human–Land Coordination Area, population and construction land grew at a rate of 0.85, which is in line with the scaling law suggesting a coordinated human–land relationship. These towns, small in number, are spatially dispersed and not located around prefecture-level cities, which indirectly reflects the land constraint and the extensive land use pattern in the vicinity of prefecture-level cities. Towns of this type may simply follow the current development path.

## 4. Discussion

In view of the great internal differences in the development level of the Loess Plateau, it was found that the Rank-Size rule and the Allometric model reveal the hierarchical structure of population size and construction land quantity as well as the characteristics of the allometric scaling evolution and contribute to a more comprehensive understanding of the relationship between population and construction land use in the Loess Plateau. The results contribute to optimizing the consolidation of construction land as well as promoting the development of a coordinated relationship between humans and land.

Firstly, the urban system is a complex regional system with a scaling phenomenon. The traditional mathematical analysis method is mainly based on the characteristic scale. The Allometric Scaling Law can effectively and quantitatively describe the spatial distribution, hierarchical structure and dynamic evolution of the urban system. Some scholars have confirmed the allometric growth relationship between population and construction land from the urban scale [40,41,42,43,44,45,46], but there is little analysis on the town scale. Lv et al. [47] and Hong et al. [49] confirmed that there is a scaling phenomenon at the town scale. This study further confirms this result that population and construction land also have scaling phenomena on the town scale. This is because different towns are organizations with self-similar structures, and towns of different sizes are actually the result of scaling. However, we also found that the results of the allometric scaling relationship were not robust between different zones. Further, we found that the results of the allometric scaling relationship of a single town changed more. Over time, the hierarchy of town-level population size in the Loess Plateau has changed from scattered to centralized, and it has been gradually rationalized. Construction land expands rapidly under the process of urbanization, and the hierarchy of construction land quantity tends to be scattered. On the whole, the change in the quantity of construction land is faster than that of population, which is the reason why the relative growth rate of construction land in most towns is faster than that of population size in the allometric analysis. Consistent with existing studies [51], the characteristics of the allometric stages of the three years are obvious, indicating that the population–construction land allometric scaling index is only constant in a certain period of time, and there must be jump characteristics in a long period of time, indicating that the relative development speed of each town is different in different stages due to different resource endowments, geographical conditions and development opportunities.

Secondly, this paper puts forward the zones of construction land consolidation and verifies the rationality of this zoning through the actual investigation results of the author in the Loess Plateau. Based on the comprehensive evaluation method, the existing studies have zoned the rural construction land consolidation on a regional scale, Li et al. [33] selected natural, demographic, transportation, economic and other factors to evaluate and concluded that the Xihaigu area of Ningxia is a high consolidation potential area. The reason is that although the natural environmental conditions in the Xihaigu area are poor, the actual survey found that the contradiction between population and construction land is not prominent in recent years. So, the comprehensive evaluation method considering natural and other factors may overestimate this result. Zhang et al. [39] concluded that Qinghai is the most restricted zone for construction land consolidation, and Gansu and Shanxi are the restricted zones for construction land consolidation. While, after considering the “nonlinear” characteristics of population and construction land, the consolidation types of construction land in Qinghai and Gansu are mostly low and medium consolidation potential areas. The difference between these results lies in the theoretical value between population and construction land not being clearly recognized, which leads to inconformity with the actual situation. The existing comprehensive evaluation methods have the problem of overestimating or underestimating the actual consolidation potential of construction land.

Thirdly, the continuous and rapid growth of construction land has created a series of human–land disharmony problems; the significance of this study is to put forward the corresponding consolidation direction of construction land based on the “non-linear” evolution law of population and construction land over a long period of time. This paper holds that the difference between the scaling coefficients of population size and construction land quantity is affected by geographical location, resource endowment, public policy, and other factors. On the whole, the expansion rate of construction land is greater than the rate of population growth (decline), and more attention should be focused on high consolidation potential areas. (1) The Southeast and Northern Shanxi Area and the Lvliang Mountain in the Eastern Gansu and Northern Shaanxi Area should be mainly concerned. The typical characteristics are the continuous population outflow and the rapid growth of construction land. The areas have serious abandonment of cultivated land caused by population loss, and the cultivated land increased by the rural residential area consolidation project will not be used. Therefore, with continuous urbanization, the construction land should be carefully converted into cultivated land, it could be naturally restored to woodland, grassland and so on for ecological functions [52]. (2) Focus on the Shanxi–Shaanxi–Inner Mongolia Contiguous Area, it is characterized by the growth of population and construction land, but the growth of construction land is still faster than the growth of population. Although the construction land still needs more increment, it needs to mainly tap the internal potential, and actively use the policy of linking urban and rural increase and decrease to provide a certain amount of high-quality increment land. (3) The population and construction land in areas with various Prefecture Level Cities as the core, such as Taiyuan, Xi’an, Luoyang and Lanzhou, have also shown a trend of growth. Over the past 20 years, land urbanization has continued to expand [53], especially around large cities. This type of area should pay attention to the intensive and economical use of construction land.

The shortcomings of this study are that due to the limitation of data at the town scale, only three years are selected and the analysis is only up to 2017. So, the error range of parameters is large. Nevertheless, statistical analysis is a confidence statement, not an absolute conclusion. Future research could conduct further exploration at the county level in consecutive years to provide a useful perspective for the theoretical development of the whole-region comprehensive land consolidation.

## 5. Conclusions

Based on the Rank-Size distribution and the theory of Allometry, this paper quantifies the human–land coordination state from two perspectives of the town hierarchic system and the growth of a single town. Based on the results of the human–land relationship, the potential zoning results of construction land consolidation in the Loess Plateau are proposed, and the corresponding consolidation direction is proposed.

(1) The majority of the towns in the Loess Plateau have small and medium-sized populations, and 62% of the towns in this area experience a reduction in population size. Geographically, the overall distribution pattern of the population sizes is “small in the north and large in the south”, and the population is concentrated in the Valley Plain; 96% of the towns show an increment in construction land, and the distribution pattern of construction land quantity is “large in the south and north but small in the middle”; the construction land is concentrated in the Valley Plain and Sandy and Desert Area.
(2) This paper confirms that the rank-size distributions of both population and construction land comply with the power-law relation. The whole town hierarchic system of the Loess Plateau is gradually tending to the optimal distribution, there is evidence of the transition of the population hierarchy from equilibrium to concentration, that is, the population tends to concentrate in high-level towns. Contrarily, the hierarchy of construction land shows an opposite pattern. The Zipf coefficient of construction land is always greater than the Zipf coefficient of the population, indicating that construction land is more concentrated than the population. What is more, this result is significantly different in each geographical zone; the zone of Valley Plain has the most balanced distribution of population and construction land; the town hierarchic system has not yet formed an ideal sequence structure.
(3) Based on the allometric scaling coefficient of population and construction land, 10 allometric types are divided. The major allometric type is positive allometry. From the results of cross-sectional allometry, the population-construction land relationship of the town system hierarchic on the Loess Plateau obeys the allometric scaling law for a long time, and the allometric scaling coefficient is in a reasonable range, which means that the human–land relationship tends to be coordinated, and the town system tends to be reasonable. However, this result is not stable in different geographical zones, especially in the zone of Irrigated Agricultural Area, where the human–land relationship is the most uncoordinated. From the results of longitudinal allometry, 90% of the town population and construction land are weakly coordinated. The reason is that the impact of the natural environment is long-term and stable, and the changes that do not follow the allometric scaling law are mainly related to socio-economic factors.
(4) The consolidation potential of construction land is divided into five types: High Consolidation Potential Area I, High Consolidation Potential Area II, Medium Consolidation Potential Area, Low Consolidation Potential Area, and Human–Land Coordination Area. The overall spatial feature of potential areas is “high in the east and low in the west”. According to the spatial distribution of different types, the corresponding suggestions for the consolidation of construction land are put forward.

## Figures and Tables

**Table 1:** Allometric levels of population and construction land.

Type	Criteria	Allometric Levels	Description	Consolidation Zone
Positive Allometry	b<−3	Population Shrinkage Positive Allometry III	The relative growth rate of construction land is much higher than the relative decline rate of the population	High Consolidation Potential I
	−3<b<−1	Population Shrinkage Positive Allometry II	The relative growth rate of construction land is higher than the relative decline rate of the population	High Consolidation Potential I
	−1<b<−0.85	Population Shrinkage Positive Allometry I	The relative growth rate of construction land is less high than the relative decline rate of the population	High Consolidation Potential I
Negative Allometry	−0.85<b<−0.5	Population Shrinkage Negative Allometry I	The relative decline rate of the population is less high than the relative growth rate of construction land	Medium Consolidation Potential
	−0.5<b<0	Population Shrinkage Negative Allometry II	The relative decline rate of the population is higher than the relative growth rate of construction land	Medium Consolidation Potential
	0<b<0.5	Population Growth Negative Allometry II	The relative growth rate of the population is higher than the relative growth rate of construction land	Low Consolidation Potential
	0.5<b<0.85	Population Growth Negative Allometry I	The relative growth rate of population is coordinated with the relative growth rate of construction land	Human–land Coordination
Positive Allometry	0.85<b<1	Population Growth Positive Allometry I	The relative growth rate of construction land is coordinated with the relative growth rate of the population	Human–land Coordination
	1<b<3	Population Growth Positive Allometry II	The relative growth rate of construction land is higher than the relative growth rate of the population	High Consolidation Potential II
	3<b	Population Growth Positive Allometry III	The relative growth rate of construction land is much higher than the relative growth rate of the population	High Consolidation Potential II

**Table 2:** Results of goodness-of-fit test of allometry of population-construction land, 2000–2017.

Year	The Loess Plateau	Valley Plain	Gullied Loess Plateau	Loess Hilly and Gully Area	Irrigated Agricultural Area	Rocky Mountain Area	Sandy and Desert Area
2000	1.4404	1.3845	1.3912	1.1682	0.6321	1.3642	0.7999
2010	1.1644	1.0429	1.331	0.9752	0.3828	0.9671	0.8551
2017	0.9666	0.9696	1.2688	0.6754	0.3007	0.8350	1.058

## Data Availability

As the data gathered came from surveys conducted by the research team, it is not publicly available.
